# Physical and Psychological Features During Remission From Non‐Specific Neck Pain

**DOI:** 10.1002/ejp.70365

**Published:** 2026-08-29

**Authors:** Cho Wai Geoffrey Yu, Kanya Wongwitwichote, Janet Deane, Valter Devecchi, Michael Mansfield, Deborah Falla

**Affiliations:** ^1^ Centre of Precision Rehabilitation for Spinal Pain, School of Sport, Exercise and Rehabilitation Sciences University of Birmingham Birmingham UK

**Keywords:** biomechanics, cervical spine, chronic pain, electromyography, recurrence

## Abstract

**Background:**

There is limited evidence on the physical and psychological features present during remission from non‐specific neck pain, yet this may be relevant for the high incidence of neck pain recurrence.

**Methods:**

A cross‐sectional study design was conducted with 60 participants (20 per group: healthy controls, people in remission from recurrent neck pain, current chronic neck pain) who were assessed for pressure pain thresholds, conditioned pain modulation, cervical kinematics, proprioception, neck muscle strength, neck muscle activity via electromyography, force steadiness, endurance, and psychological features.

**Results:**

People in remission and those with current neck pain demonstrated reduced maximal velocity (*p ≤* 0.05) and smoothness of movement (*p* ≤ 0.05) during neck extension, rotation, and side flexion compared to controls. Shoulder shrug strength (*p* ≤ 0.001) was also reduced in both pain groups. Maximal angular velocity and smoothness of neck flexion movement, as well as peak neck extension strength, were reduced only in those in remission compared to controls (*p* ≤ 0.01). Those with current neck pain distinctively showed reduced neck flexion strength, reduced force steadiness during submaximal neck flexion and extension contractions, heightened antagonist coactivity, and less endurance for the neck flexors and extensors (*p* ≤ 0.05).

**Conclusion:**

Individuals in remission from neck pain exhibit some alterations in cervical kinematics, motor output and neuromuscular control resembling similar impairments to those with current chronic neck pain despite being pain‐free. Such features may be relevant for pain recurrence, and this should be explored in future longitudinal studies.

**Significance Statement:**

This study identifies changes in physical function of the neck which are present in people with active chronic non‐specific neck pain that are also evident during remission from pain. Such findings provide an understanding of the previously underexplored clinical presentation during neck pain remission. If determined to be relevant, specific interventions to target these changes could be provided to optimise functional capacity with the aim of minimising or preventing the recurrence of pain.

## 1 Introduction

The recurrence of neck pain is common, such that approximately 23%–48% of the general population with a history of neck pain will experience a new episode within one year (Côté et al. 2004; Hill et al. 2004). Numerous adaptations to pain have been described for people with chronic neck pain including impaired motor output (e.g., reduced muscle strength and endurance) (Falla, Jull, and Hodges 2004; Schomacher and Falla 2013; Sidiropoulos et al. 2025), altered neck movement (Hwang et al. 2024; Tsang et al. 2014; Unal et al. 2024), reduced force steadiness (English et al. 2025) and increased pain sensitivity (e.g., increased mechanical sensitivity) (Johnston et al. 2008; Xie et al. 2021). Although these changes are thought to be beneficial in the short‐term to protect the painful region, some of these factors may persist, become maladaptive, and potentially contribute to the high incidence of pain recurrence (Falla and Hodges 2017; Hodges and Tucker 2011).

Psychological features are also common in people with neck pain, including passive coping, low self‐efficacy, anxiety and depression, kinesiophobia and pain catastrophising (Ahmed et al. 2019; Anarte‐Lazo et al. 2024; Andersen et al. 2016; Devecchi et al. 2022; Mercado et al. 2005; Wenzel et al. 2002). Recent cohort studies have revealed that different physical and psychological factors present during previous neck pain episodes may affect the prognosis of future episodes including perceived recovery, pain and disability (Verwoerd et al. 2024; Wingbermühle et al. 2023). Moreover, some studies have identified that various physical and psychological factors in pain‐free individuals are risk factors for future neck pain (Croft et al. 2001; Hush et al. 2009) and other work showed that depression (Bohman et al. 2019) and altered neck kinematics (Sarig Bahat et al. 2018; Weigl et al. 2021) are associated with less favourable treatment response.

Although there is an abundance of literature supporting the presence of these various physical and psychological changes accompanying active neck pain, there is limited understanding on whether such changes persist despite remission from symptoms. Only one study on people with whiplash associated disorders (WAD) showed that patients in remission from pain have greater kinesiophobia, lower quality of life, slower and irregular neck movements, and lower neck flexion strength; findings which were similar to those with chronic neck pain (Alalawi et al. 2022). Additionally, the same study showed that higher perceived disability and lower neck flexion strength during remission from pain were associated with neck pain recurrence (Alalawi et al. 2022). Thus, there is limited evidence on the physical and psychological profile of individuals in remission from non‐specific neck pain, despite this being the most prevalent subtype of neck pain (Blanpied et al. 2017). Furthermore, current literature is largely limited to neuromuscular and kinematic outcomes, with limited evaluation of other features such as conditioned pain modulation (CPM) or pressure pain threshold (PPT) assessed via quantitative sensory testing (Hübscher et al. 2013). In contrast, some evidence suggests that CPM is impaired in people with recurrent low back pain and this alteration persists during remission (McPhee and Graven‐Nielsen 2019). Whether a comparable pattern exists in people with recurrent neck pain (RNP) remains unexplored.

Addressing these gaps is clinically meaningful; if physical, neuromuscular, sensory, or psychological alterations persist during remission, they may represent modifiable targets for intervention aimed at preventing recurrence of pain which is a major unmet clinical need. Therefore, this study examined a range of physical and psychological features in people during remission from neck pain and compared their examination findings to people with current chronic neck pain (CNP) and asymptomatic controls. It was hypothesised that those in remission would show features similar to those with CNP and therefore different from asymptomatic controls.

## 2 Methods

### 2.1 Participants and Settings

This cross‐sectional study was conducted from May 2024 to March 2025. The sample size was calculated based on the study by Alalawi et al. (2022), which reported between‐group differences in neuromuscular control among healthy controls, people with chronic WAD and people with recurrent neck pain following a whiplash injury. We considered a range of outcomes of interest, including neck range of motion (ROM), neck muscle activation and strength. Because combined flexion and extension ROM resulted in the smallest effect size (Cohen's *f* = 0.42) across the outcomes of interest, the study was powered on that outcome. Using one‐way analysis of variance (ANOVA), *α* = 0.05 and power = 0.80, a total sample size of 60 participants (20 per group) was required. Sample size calculation was performed in G*Power 3.1.

Participants were recruited by convenience sampling from the community of Birmingham, United Kingdom. Recruitment posters were posted in various libraries, supermarkets, churches, community centres, etc. In addition, a recruitment advert was posted on online local community noticeboards and newsletters in Birmingham. Participants were categorised into three groups with comparable size (*n* = 20), that is, healthy controls, RNP, and CNP groups, based on the eligibility criteria presented in Table 1.

**TABLE 1:** Eligibility criteria.

Controls (*n* = 20)		RNP (*n* = 20)	CNP (*n* = 20)
Inclusion criteria		Neck pain of idiopathic origin Experience of ≥ 2 episodes of neck pain (lasting more than 24 h) separated by periods of remission (≥ 30 days) during the previous 12 months (Alalawi et al. 2022)	Neck pain of idiopathic origin lasting ≥ 3 months Neck Disability Index (NDI) minimum score: 10/50
		Pain intensity ≥ 2 (RNP: previous episode; CNP: current episode) on Numerical Pain Rating Scale (NPRS)	
	Adult aged 18–65		
Exclusion criteria	History of neck or shoulder injury History of neck pain that required treatment from a healthcare professional	Experience of neck pain over the last 30 days	
	Previous shoulder or spinal surgery; Fractured neck or shoulder; Presentation of neurological symptoms or deficits; Rheumatic joint disease; Cervical spinal infection; Cervical myelopathy; Cervical radiculopathy; Cervical radicular pain: Pregnancy		

Abbreviations: CNP, chronic neck pain; RNP, recurrent neck pain in remission.

Ethical Approval was granted from the University of Birmingham Ethics Committee (reference number: ERN_1914‐May2024) and the study adhered to the Declaration of Helsinki. Written consent was obtained from each participant prior to data collection. The study is reported according to the “Strengthening The Reporting of Observational studies in Epidemiology”, STROBE checklist (von Elm et al. 2007).

### 2.2 Instrumentation and Procedures

All data was collected in a laboratory at the Centre of Precision Rehabilitation for Spinal Pain, University of Birmingham, United Kingdom. Eligibility screening and data collection were conducted by two researchers who are physiotherapists with at least 6 years of clinical/research experience.

#### 2.2.1 Self‐Reported Outcomes

A customised questionnaire was used to collect demographic data and details about the history of neck pain. A Numerical Pain Rating Scale (NPRS) was used to evaluate neck pain intensity based on a scale ranging from 0 to 10 (Farrar et al. 2001). Participants with RNP were asked to report the worst and average pain intensity during their most recent neck pain episode, whereas participants with CNP were asked to report their current neck pain intensity.

**FIGURE 1:**
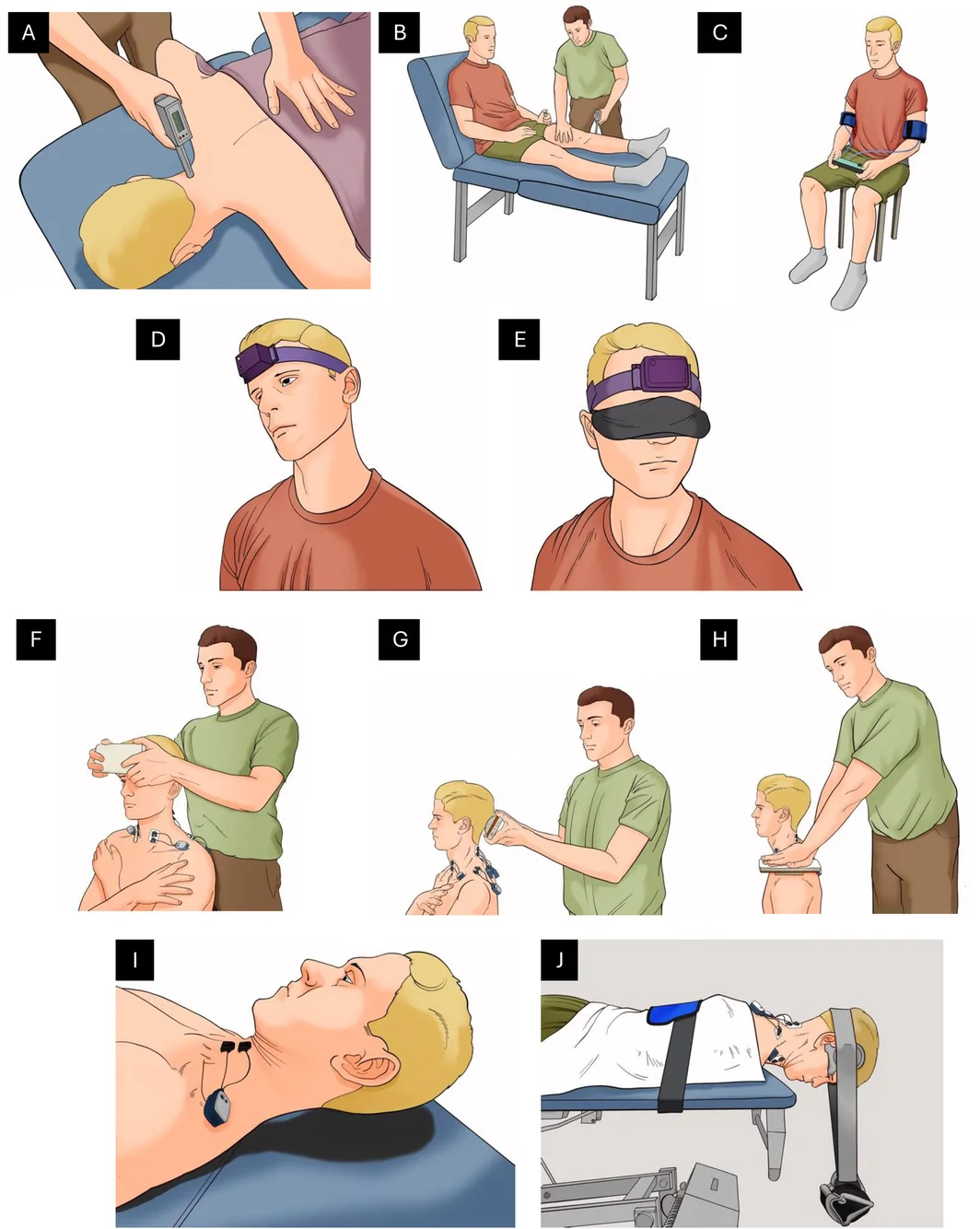
Illustrations of the experimental procedures. (A) Pressure pain algometry over a cervical facet joint; (B) Pressure pain algometry over the tibialis anterior; (C) Cuff‐pressure algometry; (D) Cervical kinematics test; (E) Cervicocephalic relocation test; (F) Cervical flexion strength test; (G) Cervical extension strength test; (H) Upper trapezius strength test; (I): Cervical flexion endurance test; (J): Cervical extension endurance test.

Before undertaking the physical measurements, all participants completed the Neck Disability Index (NDI) to assess their perceived disability due to neck pain (Vernon and Mior 1991); Tampa Scale for Kinesiophobia (TSK) to assess pain related‐fear and fear avoidance behaviours (Dupuis et al. 2023); the Pain Self‐Efficacy Questionnaire (PSEQ) to assess the optimistic self‐belief of participants with painful conditions when performing activities (Chiarotto et al. 2018); the Pain Coping Inventory (PCI) assessing three active coping strategies (pain transformation, distraction, reducing demands) and three passive coping strategies (retreating, worrying, resting) (Kraaimaat and Evers 2003); and the European Quality of Life—Visual Analog Scale (EQ‐VAS) to assess health‐related quality of life (Brooks 1996). Physical measurements were as follows and a detailed explanation is provided below: pressure pain threshold, cuff‐pressure algometry, cervical kinematics and proprioception, maximal neck muscle strength, neck muscle activity during submaximal contractions and neck flexion and extension endurance. As exercise may modify pain thresholds (Pacheco‐Barrios et al. 2020), the physical measurements started with sensory tests and followed the standardised order outlined below. The tests are illustrated in Figure 1.

##### 2.2.1.1 Pressure Pain Thresholds

PPT was tested three times with a 30‐s rest period between repetitions over the following sites: articular pillar of C2–C3 and C5–C6 facet joints, the upper trapezius (UT) midway between C7 spinous process and the acromion, and over the tibialis anterior muscle in the upper one‐third of the muscle belly to assess for widespread hyperalgesia (Lluch et al. 2014). A practice trial was conducted for each test location once prior to the actual test. A pressure algometer (Somedic, Denmark) was used with a surface area at the round tip of 1 cm^2^. Pressure was applied from the algometer probe tip at a rate of 1 kg/cm^2^ per second perpendicularly to the skin. The mean values of the three trials were taken for between group comparisons. Testing was conducted on the side of greatest neck pain for those with CNP, and the side of greatest neck pain usually experienced for those with RNP. For the control participants, neck and shoulder testing was performed on the side of the dominant arm and testing over the tibialis anterior was performed on the dominant leg.

##### 2.2.1.2 Cuff‐Pressure Algometry

A cuff pressure algometer (NociTech, Denmark) was used to measure CPM efficacy, a centrally processed measure of the net effect of the descending pain pathway. A cuff was inflated around the participant's arm, and the participant was provided with an electronic Visual Analog Scale (VAS) slider device. At the start of each trial, the participant was instructed to move the VAS slider to the right to indicate the pain intensity as soon as a painful sensation was perceived. Throughout the procedure, the participant continuously adjusted the slider to reflect their current pain intensity level. The participant was instructed to press the STOP button on the electronic VAS device when the pain tolerance limit was reached, which immediately terminates pressure application. Participants were not required to reach the maximum pain level of 10 on the scale and could stop the procedure at any point that they did not want to continue. Each participant was familiarised with the use of the VAS slider before the start of the measurement.

Using this method, the pain detection threshold (PDT) and tolerance threshold (PTT) in kPa were assessed separately on each side. PDT is defined as the lowest pressure at which the participant just notices pain and PTT is the pressure which the participant finds the pain unbearable. After measuring PDT and PTT on each arm, the cuff on the non‐dominant arm was inflated to 70% of PTT of that arm as the conditioning stimulus, while the cuff on the dominant arm was also inflated simultaneously to assess the PDT and PTT on the dominant arm. CPM efficacy was calculated as the difference in PDT and PTT values in kPa on the dominant arm between the conditioning phase (with non‐dominant arm stimulation) and the baseline measurements (without conditioning stimulus) (Yarnitsky et al. 2015).

##### 2.2.1.3 Cervical Kinematics and Proprioception

A Senscoordination 3D Cervical Trainer (Sensamove, The Netherlands) was used to assess cervical kinematics and joint position error (JPE). The SCT is a 9‐degree of freedom unit which records combined signals from a 3‐axis accelerometer, a 3‐axis gyroscope and a 3‐axis magnetometer and it translates the data to an orientation vector in *x, y* and *z* coordinates and an angle of rotation around the direction of the vector. It was positioned with an adjustable strap around the participant's head such that the sensor aligned with the centre of the forehead just above the bridge of the nose. The system was calibrated before each task. The Senscoordination 3D Cervical Trainer has high concurrent validity and inter‐rater reliability (Thoomes et al. 2023). Participants performed neck flexion/extension, right/left side flexion and rotation at a self‐selected velocity, with the instruction to reach their maximal ROM. Each movement was repeated three times.

For each movement direction, ROM, maximum angular velocity of movement and the jerk index (which reflects smoothness of movement) were measured (Devecchi et al. 2022; Sjölander et al. 2008). Raw data recorded from the Senscoordination 3D Cervical Trainer were exported and analysed offline in MATLAB (The Mathworks, R2018b, USA) using customised scripts. Data were low‐pass filtered using a 4th order Butterworth filter with a 10 Hz cut‐off frequency (Sarig Bahat et al. 2014). Using the movement's angle waveform recorded with the sampling interval (0.04 s, 25 Hz), angular velocity was calculated by differentiating the movement angle over time. For each movement waveform, positive and negative peaks respectively represented the maximal ROM in flexion/extension, right/left side flexion and right/left rotation. Considering these movement peaks, segments of full‐range movements were identified (Devecchi et al. 2022; Sjölander et al. 2008). Angular velocity was computed for each segment. Then, the average ROM and maximal angular velocity in each of the three physiological movement planes were calculated for each participant. To assess the smoothness of movement, the second derivative of the velocity‐time series was taken to compute the jerk index. By multiplying a scaling factor and applying log transformation, a dimensionless jerk index was created such that a higher jerk index indicates smoother movement (Devecchi et al. 2022; Sjölander et al. 2008).

To assess JPE, the participant was instructed to close their eyes with their head in a neutral position, then maximal right or left cervical rotation was performed at a natural speed and the participant attempted to return as accurately as possible to the neutral position. When the participant indicated that they had returned to their start location, the relocation error (in degrees) was recorded (Kristjansson et al. 2003). JPE in the transverse plane was represented by the mean absolute relocation errors in degrees from the three attempts for each direction (Treleaven et al. 2003).

##### 2.2.1.4 Maximal Neck Muscle Strength

A NOD dynamometer (OT Bioelettronica SRL, Turin, Italy) was used to assess peak force during maximal neck strength tests with electromyographic signals recorded using wireless sensors (Ultium, Noraxon, USA) with a sampling frequency of 2000 Hz and with the data converted to digital form by a 16‐bit A/D converter. The common‐mode rejection ratio was greater than 100 dB and the input impedance greater than 100 MΩ. Signals were recorded and visually checked using the software myoRESEARCH 3.12 (Noraxon, USA). Data were then exported to a hard drive for further analysis. The NOD dynamometer has been shown to be a valid tool for assessing neck muscle strength (Arvanitidis et al. 2025). The reporting of electromyography (EMG) measures and results adheres to the CEDE‐Check (Besomi et al. 2024); a checklist to assist researchers to thoroughly report their EMG methodologies.

Electromyography was recorded bilaterally from the sternocleidomastoid (SCM) to assess superficial neck flexor activity, while superficial neck extensor activity was assessed with electrodes positioned over the bilateral splenius capitis (SC) and UT. Dual electrodes with a 1‐cm diameter and a fixed 2‐cm interelectrode distance were applied. For SCM, the electrodes were placed at the midpoint between the mastoid process of occiput and sternal insertion over the muscle belly (Falla et al. 2002), for SC, the electrodes were placed 2 cm lateral to the C4 spinous process bilaterally (Sommerich et al. 2000); and for the UT, the electrodes were placed at 50% on the line from the acromion to C7 (Mathiassen et al. 1995). To decrease skin impedance, the test area was shaved when necessary, and then scrubbed with an exfoliating paste (SPES Medica, Italy) and then washed and dried prior to electrode placement.

Maximal isometric neck and shoulder strength tests were conducted following the order of neck flexion, neck extension and finally bilateral shoulder shrug. Each 5 s isometric maximal voluntary contraction (MVC) was performed twice with a minute of rest between repetitions. During neck flexion and extension testing, participants sat on a chair with a waist strap to prevent compensatory upper body movements. For neck flexion testing, the NOD dynamometer was positioned above the eyebrows, whereas for extension testing, it was placed against the back of the head at the occiput. The participant performed two maximum isometric contractions against the NOD dynamometer in each of the two directions. Shoulder shrug strength was measured with participants seated upright, arms positioned by their sides with forearms extended and palms facing inward. The NOD dynamometer was positioned over the participant's acromion process by the investigator, who provided manual resistance while the participant performed maximum voluntary shoulder elevation contractions. For all MVC test positions, participants performed one practice trial with a 5 s submaximal contraction at ~50% of maximal effort to familiarise themselves with the procedure. For the actual test attempts, verbal encouragement was given to facilitate participants to achieve genuine maximal effort. Peak force in Newton was recorded from the NOD dynamometer using the associated Android app (NOD app, OT Bioelettronica, Turin, Italy) and the highest value across the two repetitions was taken forward for further analysis. In the case of shoulder shrug, data was averaged across sides.

The analysis of EMG data was conducted with a customised script using MATLAB (R2023a, The Mathworks Inc., USA). EMG signals were band‐pass filtered between 20 and 400 Hz using a 4th order Butterworth filter, and a 50‐Hz notch filter was applied (Park et al. 2013; Troiano et al. 2008). All EMG signals and power spectrums were visually inspected for the presence of artefacts. From the EMG signals, the maximum root mean square (RMS) value was determined from each MVC contraction, computed with a 500 ms sliding window with 400 ms overlap, and the highest value across the two repetitions was taken forward for further analysis. Antagonist muscle activity was determined as the normalised RMS relative to the maximal RMS value measured during the MVC when the muscle was acting as an agonist. For example, SCM antagonist coactivity during the neck extension MVC contraction was determined by normalising the average RMS during neck extension by the peak RMS measured during the neck flexion MVC test and expressing it as a percentage (Florencio et al. 2015).

##### 2.2.1.5 Submaximal Contractions

In a seated position, participants then performed one repetition of submaximal neck flexion and extension isometric contractions at 20% MVC, each for 30 s. Visual feedback on force was displayed on the tablet and the participants were instructed to hold the contraction as steady as possible. Force signals were low‐pass filtered using a third‐order Butterworth filter at 15 Hz and force steadiness was assessed in absolute terms using the standard deviation (SD) and relative terms using the coefficient of variation (CoV). The smaller the SD and CoV are, the steadier the contractions are.

##### 2.2.1.6 Neck Flexion and Extension Endurance

Neck flexion endurance was tested first with the participant positioned in supine on the examination table with their neck in a neutral position and their knees and hips flexed to 90 degrees. The instruction was to “nod the chin slightly and lift the head away from the table and hold it as long as you can”. Verbal encouragement was given every 30 s to prompt the participant to maintain the hold. Task failure was defined as the inability to hold the craniocervical flexion component for more than 5 s or inability to keep the head above the examination table despite verbal encouragement (Halvorsen et al. 2016).

Five minutes after the flexion endurance test, the neck extension endurance test was conducted in a prone position with a strap around the participant's trunk at the level of T7. The participant's head and neck were positioned away from the table, a 2 kg suspended load was placed on the head, and they were asked to hold the head and neck position steady with the neck in a neutral position for as long as possible. Verbal encouragement was given every 30 s to prompt the participant to maintain the hold. An inclinometer, embedded within an elastic headband, was placed around the participant's head just above the ear and the reading was adjusted to be 0 degrees at the start position. Task failure was defined as movement of the head and neck by five or more degrees despite verbal encouragement (Peolsson et al. 2007).

For both flexion and extension endurance tests, the time to task failure was recorded and myoelectric manifestations of muscle fatigue were assessed using the median frequency (MDF) of the agonist muscles (Falla et al. 2003). Specifically, the slope of MDF of the EMG signal was determined for SCM and SC for flexion and extension endurance tests respectively. The MDF was calculated within non‐overlapping 2‐s epochs across the duration of the sustained contractions followed by linear regression to determine the rate of change of MDF (Merletti et al. 2008). The data were normalised to the regression line's starting point (intercept), and the slope served as the regression coefficient for between‐group comparisons (Falla, Jull, Rainoldi, and Merletti 2004).

### 2.3 Statistical Analysis

Statistical analysis was conducted using IBM SPSS Statistics, Version 29.0, 2023. Histograms and QQ plots were used for visual inspection of the presence of outliers. The distribution of the data for each variable and group was analysed with the Shapiro–Wilk test. Homogeneity of variance for each feature was assessed using the Levene's test. Descriptive statistics are reported using mean with SD for parametric data and median with interquartile range (IQR) for non‐parametric data.

Chi‐Square test was used to examine if the gender ratio was similar between groups with the alpha level set at 0.05. ANOVA was used to investigate differences across groups for each measure. In case of significant findings for the ANOVA (*α* < 0.05), post hoc analysis was conducted and reported after adjusting for Bonferroni's correction considering three between‐group comparisons with an adjusted alpha level set at 0.05. When data were not normally distributed or heterogeneity of variance was found, the Kruskal–Wallis test was used; when the result was significant (*α* < 0.05), between‐group comparisons were assessed by multiple Wilcoxon rank‐sum tests with an adjusted alpha level set at 0.05 using Bonferroni's correction.

## 3 Results

### 3.1 Participant Characteristics

Demographic characteristics of the participants are reported in Table 2. The mean age was 30.2 ± 8.1 years for the healthy controls, 33.3 ± 8.7 years for those with CNP, and 33.6 ± 8.1 years for those with RNP (*p* > 0.05). The majority of the participants were females in all three groups (*p* = 0.377). No significant differences were observed for participant demographics across groups. The mean score of average neck pain intensity for those with CNP during the current episode and those with RNP for their most recent pain episode was the same (5.4 ± 1.5).

**TABLE 2:** Baseline characteristics of the participants (mean ± standard deviation (SD)).

Baseline characteristics	Controls (*n* = 20)	RNP (*n* = 20)	CNP (*n* = 20)	*p*‐value (one‐way ANOVA)
Gender (M:F)	9:11	5:15	6:14	0.377
Age	30.2 ± 8.1	33.6 ± 8.1	33.3 ± 8.7	0.364
BMI	23.5 ± 4.0	22.6 ± 3.1	25.1 ± 6.0	0.312
Current NPRS (0–10)	0	0	5.4 ± 1.5	/
NDI (0–50)	1.1 ± 1.5	7.3 ± 5.0	14.6 ± 5.8	< 0.01**
Headache score from the NDI (0–5)	0.2 ± 0.5	1.0 ± 0.8	1.7 ± 1.5	/
Number of neck pain episodes in recent 12 months	/	7.0 ± 5.6	/	/
Average pain (last episode)	/	5.4 ± 1.5	/	/
Worst pain (last episode)	/	7.2 ± 1.4	/	/

Abbreviations: BMI, body mass index; NDI, neck disability index; NPRS, numeric pain rating scale.

** Statistically significant difference for all post hoc pairwise comparison.

### 3.2 Sensory Function

PPT over the C2/3 and C5/6 facet joints, UT and tibialis anterior did not differ across groups (*H* ≤ 3.227, *p* ≥ 0.199). There was no significant difference (*H* = 0.577, *p* = 0.623) in CPM efficacy of PTT. For CPM efficacy of PDT, there were group differences based on the ANOVA (*F* = 3.379, *p* = 0.041), but no significant difference was found in post hoc pairwise comparison (*p* ≥ 0.053). Although post hoc comparisons were not statistically significant, mean CPM efficacy of PDT was lower in both neck pain groups than in controls. It should be noted that nine of the 20 control participants withstood the maximum pressure allowed by the cuff‐pressure algometer in both PTT measurements, which created a ‘ceiling effect’ such that the true PTT CPM efficacy could not be reflected in these participants. In contrast, all participants in both CNP and RNP groups had PTT lower than the maximum pressure of the device in all measurements (Table 3).

**TABLE 3:** Descriptive statistics (mean ± standard deviation (SD) or median and interquartile range (IQR)) and between‐group comparison for sensory measurements.

Outcomes	Controls	RNP	CNP	*F* or *H*‐statistics and overall *p*‐value	Post hoc *p*‐value (Controls vs. RNP)	Post hoc *p*‐value (Controls vs. CNP)	Post hoc *p*‐value (RNP vs. CNP)
*Pressure pain threshold* (kPa)							
C2/3 facet joint	214.3 (178.0–317.9)	285.5 (231.8–286.2)	218.5 (182.0–349.3)	*H* = 1.115 *p* = 0.573	—	—	—
C5/6 facet joint	231.0 (174.2–319.8)	239.8 (218.3–293.3)	237.7 (165–313.6)	*H* = 0.170 *p* = 0.919	—	—	—
Upper trapezius	316.2 (232.3–441.0)	296.8 (252–340.9)	284.5 (167.6–380.3)	*H* = 1.015 *p* = 0.602	—	—	—
Tibialis anterior	500.7 (338.3–578.0)	428.8 (352.0–535.8)	357.0 (274.3–454.4)	*H* = 3.227 *p* = 0.199	—	—	—
*Conditioned pain modulation* (kPa)							
CPM efficacy (pain detection threshold)	8.1 ± 8.7	1.8 ± 9.0	3.0 ± 6.6	*F* = 3.379 *p* = 0.041*	0.053	0.156	1.000
CPM efficacy (pain tolerance threshold)	0 (−0.7–5.3)	−1.5 (−4.1–9.2)	3.5 (−4.0–8.6)	H = 0.577 *p* = 0.623	—	—	—

Abbreviations: CNP, chronic neck pain; CPM, conditioned pain modulation; RNP, recurrent neck pain in remission.

* *p* < 0.05 for the overall statistical significance in one‐way ANOVA.

### 3.3 Kinematics and Proprioception

**FIGURE 2:**
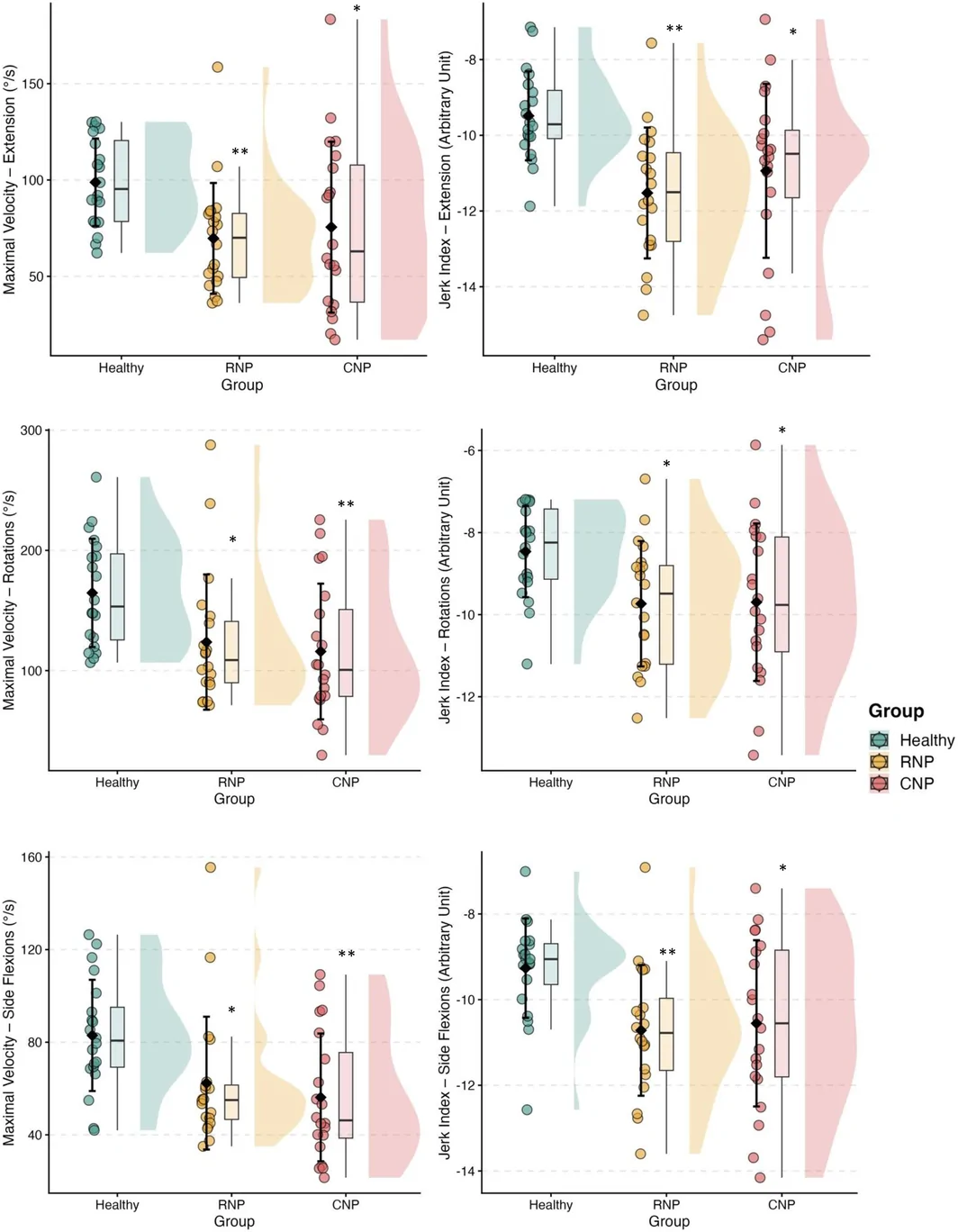
Results presenting kinematic measurements where a significant difference was observed in both the comparison of the chronic pain group with the control participants and the comparison of recurrent neck pain in remission group with the control participants. The distributions are illustrated with half‐violin plots accompanied by embedded boxplots and individual data point. The mean is displayed as a black diamond with error bars representing ±SD. *p < 0.05 in pairwise comparison with healthy participants in post‐hoc analysis with Bonferroni's correction. **p < 0.01 in pairwise comparison with healthy participants in post‐hoc analysis with Bonferroni's correction.

Participants with CNP and RNP showed similar ROM for all movement directions compared to healthy controls (Table 4). However, both the CNP and RNP groups showed reduced maximal angular velocity of cervical extension (*p* ≤ 0.049), bilateral rotation (*p ≤* 0.013), and bilateral side flexion (*p ≤* 0.033). Additionally, both the CNP and RNP groups showed decreased smoothness of movement compared with healthy controls for the movements of cervical extension (*p* ≤ 0.044), bilateral rotation (*p ≤* 0.045) and bilateral side flexion (*p ≤* 0.048). For maximal angular velocity and smoothness of movement during cervical flexion, only the RNP group showed significant differences to healthy controls (*p ≤* 0.004). Figure 2 presents the kinematic measurements which were different for those with either CNP or RNP versus healthy controls.

**TABLE 4:** Descriptive statistics (mean ± standard deviation (SD) or median and interquartile range (IQR)) and between‐group comparison for cervical kinematics and sensorimotor control measurements.

Outcomes	Controls	RNP	CNP	*F* or *H*‐statistics and overall *p*‐value	Post hoc *p*‐value (Controls vs. RNP)	Post hoc *p*‐value (Controls vs. CNP)	Post hoc *p*‐value (RNP vs. CNP)
**Kinematics**							
*Range of motion* (°)							
Flexion	59.8 ± 12.4	60.5 ± 13.1	54.7 ± 10.5	*F* = 1.268 *p* = 0.289	—	—	—
Extension	61.6 ± 13.0	67.4 ± 13.9	55.4 ± 15.0	*F* = 3.648 *p* = 0.032*	0.582	0.513	0.027**
Left and right rotation	134.7 (126.1–139.8)	134.6 (129.6–151.2)	133.0 (114.3–142.8)	*H* = 1.657 *p* = 0.437	—	—	—
Left and right side flexion	83.6 ± 13.9	86.89 ± 16.5	74.8 ± 16.0	*F* = 3.232 *p* = 0.139	—	—	—
*Maximal velocity* (°/s)							
Flexion	98.8 (08.6–114.4)	57.7 (51.7–84.1)	74.3 (36.0–127.1)	*H* = 10.229 *p* = 0.006*	0.004**	0.172	0.605
Extension	95.3 (78.2–123.6)	70.0 (48.0–83.2)	63.0 (35.6–111.0)	*H* = 10.371 *p* = 0.006*	0.007**	0.049**	1.000
Left and right rotation	153.3 (121.6–201.1)	134.6 (129.6–151.2)	100.6 (77.5–158.4)	*H* = 11.726 *p* = 0.003*	0.013**	0.007**	1.000
Left and right side flexion	80.7 (68.8–99.2)	55.0 (45.6–62.4)	46.2 (36.1–81.2)	*H* = 11.361 *p* = 0.003*	0.033**	0.004**	1.000
*Jerk index‐smoothness of movement* (a.u.)							
Flexion	−10.1 ± 1.5	−12.1 ± 1.6	−11.1 ± 2.0	*F* = 6.474 *p* = 0.003*	0.002**	0.207	0.260
Extension	−9.7 (−10.2 to −8.7)	−11.5 (−12.9 to −10.3)	−10.5 (−11.9 to −9.7)	*H* = 14.667 *p* < 0.001*	< 0.001**	0.044**	0.550
Left and right rotation	−8.5 ± 1.1	−9.7 ± 1.5	−9.7 ± 1.9	*F* = 4.335 *p* = 0.018*	0.037**	0.045**	1.000
Left and right side flexion	−9.1 (−9.9 to −8.7)	−10.8 (−11.7 to −9.5)	−10.6 (−11.8 to −8.8)	*H* = 10.899 *p* = 0.004*	0.005**	0.048**	1.000
**Sensorimotor control**							
*Joint position error* (°)							
Left	2.0 (1.1–2.9)	2.1 (1.3–4.4)	2.1 (1.1–4.9)	*H* = 1.105 *p* = 0.576	—	—	—
Right	2.2 (1.7–3.2)	1.7 (1.7–5.1)	2.5 (1.5–4.4)	*H* = 1.171 *p* = 0.557	—	—	—

Abbreviations: a.u., arbitrary unit; CNP, chronic neck pain; RNP, recurrent neck pain in remission.

* *p* < 0.05 for the overall statistical significance in one‐way ANOVA or Kruskal–Wallis test.

** *p* < 0.05 for post hoc analysis with Bonferroni's correction.

JPE for both right and left rotation did not differ across groups (*H* ≤ 1.171, *p* ≥ 0.557).

### 3.4 Motor Output and Neuromuscular Control

The results for all measurements are presented in Table 5. The average peak force of shoulder shrug from the two sides was also less in both the CNP and RNP groups (*p* ≤ 0.001). Only the CNP group showed lower flexion peak force (*p* = 0.038) and only the RNP group (*p* = 0.008) showed lower extension peak force compared with healthy controls.

**TABLE 5:** Descriptive statistics (mean ± standard deviation (SD) or median and interquartile range (IQR)) and between‐group comparison for motor output and measures of muscle activity.

Outcomes	Controls	RNP	CNP	*F* or *H*‐statistics and overall *p*‐value	Post hoc *p*‐value (Controls vs. RNP)	Post hoc *p*‐value (Controls vs. CNP)	Post hoc *p*‐value (RNP vs. CNP)
**Motor output**							
*Peak force* (N)							
Flexion	70.8 (55.3–112.0)	56.5 (43.0–65.9)	53.5 (25.6–71.6)	*H* = 7.735 *p* = 0.021*	0.061	0.038**	1.000
Extension	101.3 (84.1–108.9)	81.0 (68.4–89.5)	85.0 (56.1–115.1)	*H* = 9.198 *p* = 0.010*	0.008**	0.183	0.779
Shoulder shrug (side‐averaged)	226.8 (190.6–334.4)	161.0 (120.1–184.5)	145.0 (96.4–231.5)	*H* = 18.268 *p* < 0.001*	< 0.001**	0.001**	1.000
*Antagonist coactivity* (%)							
SCM	5.3 (4.1–17.5)	15.2 (9.6–33.7)	15.2 (9.3–25.5)	*H* = 8.704 *p* = 0.013*	0.085	0.015**	1.000
SC	23.7 (15.9–32.9)	31.4 (22.9–48.6)	36.3 (30.6–48.3)	*H* = 9.573 *p* = 0.008*	0.183	0.006**	0.696
*Force steadiness—coefficient of variation* (a.u.)							
Flexion 30 s	3.2 (2.2–4.2)	3.2 (2.7–5.6)	4.7 (3.9–4.9)	*H* = 6.849 *p* = 0.033*	0.795	0.027**	0.405
Extension 30 s	3.3 (2.6–4.1)	3.7 (2.8–4.1)	4.9 (3.3–6.1)	*H* = 9.707 *p* = 0.008*	0.634	0.006**	0.194
*Force steadiness—standard deviation* (V)							
Flexion 30 s	0.5 (0.4–0.9)	0.5 (0.5–0.8)	0.8 (0.7–0.9)	*H* = 7.065 *p* = 0.029*	0.175	0.031**	1.000
Extension 30 s	0.6 (0.5–0.7)	0.6 (0.5–0.7)	0.8 (0.6–0.9)	*H* = 8.555 *p* = 0.014*	0.060	0.021**	1.000
*Endurance‐time to failure (s)*							
Flexion	63.0 (45.6–91.7)	47.7 (19.0–68.2)	34.3 (24.7–51.7)	*H* = 9.671 *p* = 0.008*	0.075	0.008**	1.000
Extension	240.0 (240.0)	240 (115.4–240.0)	192.5 (123.5–240.0)	*H* = 6.372 *p* = 0.041*	0.251	0.042**	1.000
*Slope of the median frequency*							
Flexion	−0.2 (−0.3 to −0.1)	−0.3 (−0.5 to −0.1)	−0.32 (−0.39 to −0.04)	*H* = 2.137 *p* = 0.344	—	—	—
Extension	0 (−0.01–0.01)	−0.01 (−0.03–0)	0 (−0.01–0.01)	*H* = 1.860 *p* = 0.395	—	—	—

Abbreviations: a.u., arbitrary unit; CNP, chronic neck pain; RMS, root mean square; RNP, recurrent neck pain in remission; SD, standard deviation.

* *p* < 0.05 for the overall statistical significance in one‐way ANOVA or Kruskal–Wallis test.

** *p* < 0.05 for post hoc analysis with Bonferroni's correction.

During maximal isometric neck flexion, the antagonist coactivity of SC was significantly greater in the CNP group compared with controls (*p* = 0.006). For maximal isometric extension, the antagonist coactivity of SCM was also significantly greater in the CNP group compared with healthy controls (*p* = 0.015).

For the 30‐s isometric neck flexion and extension contractions performed at 20% MVC, only the CNP group had less force steadiness (higher CoV and SD) compared to the healthy controls (*p* ≤ 0.031).

Only the CNP group had a shorter time to failure compared to healthy controls during both the sustained neck flexion and extension endurance tests (*p* ≤ 0.042). The rate of change of MDF was not different across groups for either task (*H* ≤ 2.137, *p* ≥ 0.344).

### 3.5 Psychological Features

No statistically significant between‐group difference was noted for any of the questionnaires (*p* ≥ 0.103) (Table 6). For the TSK, only the CNP group presented with a group average above the cutoff score of 37 which defines the presence of kinesiophobia (Cleland et al. 2008).

**TABLE 6:** Descriptive statistics (mean ± standard deviation (SD) or median and interquartile range (IQR)) and between‐group comparison for psychological features.

Outcomes (score range)	Controls	RNP	CNP	*F* or *H*‐statistics and overall *p*‐value
TSK (17–68)	34.80 ± 7.52	33.95 ± 6.13	37.45 ± 5.74	*F* = 1.574 *p* = 0.216
PSEQ (6–60)	49.0 (39.5–53)	51.5 (38.8–56.0)	44.0 (37.3–52.0)	*H* = 3.433 *p* = 0.180
PCI (active coping) (12–48)	33.0 (29.3–35.0)	29.0 (25.0–34.8)	31.5 (27.0–33.8)	*H* = 3.891 *p* = 0.143
PCI (passive coping) (21–84)	50.0 (43.5–55.5)	44.5 (39.0–52.8)	48.5 (44.3–59.0)	*H* = 3.525 *p* = 0.172
EQ‐VAS (0–100)	85.0 (80.0–90.0)	80.0 (70.0–90.0)	72.5 (52.5–89.3)	*H* = 4.552 *p* = 0.103

Abbreviations: CNP, chronic neck pain; EQ‐VAS, European Quality of life—Visual Analog Scale; PCI, Pain Coping Inventory; PSEQ, Patient Self‐Efficacy Questionnaire; RNP, recurrent neck pain in remission; TSK, Tampa Scale for Kinesiophobia.

## 4 Discussion

This study aimed to assess physical and psychological features in the remission phase of RNP and determine whether any features were comparable to those with CNP and different from healthy pain‐free controls. This represents the first study to characterise the physical and psychological profile of individuals in remission from non‐specific neck pain, the most prevalent subtype of neck pain, and to compare this profile directly against those with current chronic neck pain and healthy pain‐free controls. Findings suggest that the following features were similar between those in remission from RNP and those with CNP: (1) decreased maximal angular velocity and smoothness of movement in neck extension, rotation and side flexion and (2) decreased shoulder shrug strength. The maximal angular velocity and smoothness of movement of neck flexion and neck extensor strength were reduced only in those in remission from pain with respect to pain‐free controls. In addition, those with current neck pain distinctively showed reduced neck flexion strength, reduced force steadiness during submaximal cervical neck flexion and extension, heightened sternocleidomastoid and splenius capitis coactivity and less endurance for the neck flexors and extensors.

Previous studies have reported impaired neck movement velocity and smoothness in individuals with chronic neck pain (Sarig Bahat et al. 2010; Tsang et al. 2014). In the current study, although ROM did not differ across groups, the maximal velocity and smoothness of movement in multiple planes were lower for both RNP and CNP groups. This finding demonstrates that kinematic impairments persist despite remission from neck pain and include slower and less smooth neck movements, features more directly reflective of motor control. These findings may be relevant for neck pain recurrence. A previous study using video fluoroscopy reported increased variability of cervical joint motion in individuals with recurrent neck pain which suggested an alteration in kinematic control (Qu et al. 2020). Whilst neck ROM for those in remission from pain was comparable to healthy controls, they did move their neck more slowly and in a jerkier way. Since the velocity and smoothness of movement relate more to motor control than simply ROM, it is speculated that the control of movement may take longer to recover following a period of neck pain and may require specific interventions (i.e., motor control exercises) in order to gain recovery of movement control.

Reduced upper trapezius strength has been documented previously for people with chronic neck pain (Nunes et al. 2026; Park et al. 2020). In our study, both the RNP and CNP groups showed reduced average shoulder shrug strength. Critically, the persistence of this neuromuscular impairment during remission is a novel finding that suggests this is not a simply pain‐state phenomenon but may represent vulnerability that outlasts the painful episode itself.

We hypothesised that both the RNP and CNP groups would present with lower PPT and CPM efficacy compared to healthy individuals. However, the findings did not support our hypothesis. This is the first study to investigate CPM efficacy specifically in people who are in remission from neck pain. In addition, it is also the first study to investigate CPM efficacy in non‐specific neck pain using pressure as both a conditioning stimulus and test stimulus. In a recent systematic review investigating CPM in people with chronic non‐specific neck pain (Arribas‐Romano et al. 2024), it was suggested that there was impaired CPM efficacy when assessed locally in the neck region but not remotely with all five included studies using pressure as the test stimulus and cold water as the conditioning stimulus. In addition, with cuff‐pressure algometry, it was shown that CPM is impaired in people with recurrent LBP compared to healthy controls and that this alteration persists during remission (McPhee and Graven‐Nielsen 2019). In the current study, quite often participants could withstand the maximal pressure (97.3 to 98.0 kPa) for both assessments of PTT of the dominant arm with and without conditioning stimulus. This phenomenon was present in nine of the 20 healthy participants but none of the participants in both CNP and RNP groups. As a result, the actual increase of PTT (hence CPM efficacy) may not be reflected because of this ‘ceiling effect’ in these healthy participants which could possibly explain the non‐significant group differences.

The current results did not support the presence of significant psychological changes in those with RNP, which is different from a previous study which showed that people with RNP presented with greater kinesiophobia and lower quality of life with respect to healthy controls, although it should be noted that this was people in remission from pain following trauma‐induced neck pain (Alalawi et al. 2022). It should be appreciated that the contribution of biological (physical) and psychological factors in patients with musculoskeletal pain varies between individuals (Jull 2017) and it may be that some of the participants with RNP had relevant levels of psychological features e.g., five of the 20 participants in the RNP group presented with a TSK score greater than 37/68 which indicated high levels of kinesiophobia. It should also be highlighted that features such as higher levels of pain catastrophising, depression, anxiety and distress are associated with the persistence and recurrence of neck pain (Croft et al. 2001; Shahidi et al. 2015; Xie et al. 2022), although these studies did not specify if the participants were in remission at baseline or if they were free from a history of neck pain.

### 4.1 Strengths and Limitations

To the best of our knowledge, this is the first study to comprehensively characterise physical and psychological features during remission from non‐specific neck pain, incorporating neuromuscular, kinematic, sensory and psychological domains and benchmarking these features against both symptomatic people and a healthy control group. This comparison enables a better understanding of the clinical presentation of people in remission from RNP and the identification of potentially relevant physical features which may be related to the recurrent nature of their pain.

Several methodological limitations warrant consideration. Convenience sampling with a relatively small sample size may introduce potential selection bias, as the participants may not be fully representative of the broader RNP population, thereby limiting generalisability. There was a lack of blinding of the assessors for the participants' group allocation. To minimise bias, instructions were standardised for all physical measurements. It is expected that minimal if any bias could be introduced when using equipment such as movement analysis and electromyography, and the risk of bias from lack of blinding should be irrelevant for the self‐reported outcomes. Because of the case–control design, it is also difficult to interpret whether the neuromuscular changes observed preceded the onset of pain or resulted from pain. Additionally, the large number of outcome variables examined increases the risk of Type I error despite the application of Bonferroni correction, and therefore some significant findings should be interpreted cautiously until replicated in independent samples. Despite these limitations, the present design is consistent with the exploratory aims of examining physical and psychological features across groups during remission from neck pain, and the findings contribute to understanding whether individuals in remission retain characteristics more similar to those with current neck pain than to asymptomatic controls.

### 4.2 Research and Clinical Implications

Some changes in physical function of the neck which are present in people with CNP are also evident during remission from neck pain. Future longitudinal studies should assess the relevance of such features with the incidence, frequency and severity of persistent and recurrent neck pain. If determined to be relevant, specific interventions such as precise kinematic training with virtual reality (Sarig Bahat et al. 2018) and strengthening exercise (Giménez‐Costa et al. 2022; Jull et al. 2009) could then be provided to optimise functional capacity with the aim of minimising or preventing the recurrence of pain. As seen from the data, there was large individual variability in physical and psychological features between individuals in remission of neck pain, supporting the need for individualised and targeted interventions.

## 5 Conclusion

Similar to people with CNP, individuals with recurrent episodes of neck pain tested during remission from pain present with changes in several physical domains, including neck kinematics and neck strength. Future longitudinal studies are needed to investigate the relevance of these features for the recurrence of neck pain.

## Author Contributions

**Cho Wai Geoffrey Yu:** conceptualization, methodology, data collection, writing – original draft preparation; **Kanya Wongwitwichote:** data collection, writing – reviewing; **Janet Deane:** conceptualization, methodology, writing – reviewing; **Valter Devecchi:** conceptualization, methodology, writing – reviewing; **Michael Mansfield:** conceptualization, methodology, writing – reviewing; **Deborah Falla:** conceptualization, methodology, writing – reviewing. All authors discussed the results and commented on the manuscript. Deborah Falla is guarantor.

## Funding

The authors have nothing to report.

## Disclosure

Use of Artificial Intelligence: Artificial intelligence is not used for this manuscript.

## Conflicts of Interest

The authors declare no conflicts of interest.

## Supporting information


**Data S1:** STROBE Statement—Checklist of items that should be included in reports of *cross‐sectional studies*.
